# Structural Dependent Eu^3+^ Luminescence, Photoelectric and Hysteresis Effects in Porous Strontium Titanate

**DOI:** 10.3390/ma13245767

**Published:** 2020-12-17

**Authors:** Maryia Rudenko, Nikolai Gaponenko, Vladimir Litvinov, Alexander Ermachikhin, Eugene Chubenko, Victor Borisenko, Nikolay Mukhin, Yuriy Radyush, Andrey Tumarkin, Alexander Gagarin

**Affiliations:** 1 Department of Micro- and Nanoelectronics, Belarusian State University of Informatics and Radioelectronics, P. Browka Str. 6, 220013 Minsk, Belarus; nik@nano.bsuir.edu.by (N.G.); eugene.chubenko@gmail.com (E.C.); borisenko@bsuir.by (V.B.); 2 Department of Condensed Matter Physics, National Research Nuclear University MEPhI, Kashirskoe Shosse 31, 115409 Moscow, Russia; 3 Department of Micro and Nano Electronics, Ryazan State Radio Engineering University, Gagarin Str. 59, 390005 Ryazan, Russia; vglit@yandex.ru (V.L.); al.erm@mail.ru (A.E.); 4 Faculty of Electronics, Saint Petersburg Electrotechnical University “LETI”, Professor Popov Str. 5, 197376 Saint Petersburg, Russia; muhinnv_leti@mail.ru (N.M.); avtumarkin@yandex.ru (A.T.); AGGagarin@gmail.com (A.G.); 5 Department of Engineering, University of Applied Sciences Brandenburg, Magdeburger Str. 50, 14770 Brandenburg an der Havel, Germany; 6 Scientific-Practical Materials Research Centre of NASB, P. Browka Str. 19, 220072 Minsk, Belarus; radyush@ifttp.bas-net.by

**Keywords:** europium luminescence, porous film, strontium titanate, resistive switching hysteresis, photocurrent

## Abstract

Eu^3+^ doped porous nanostructured SrTiO_3_ films and powder fabricated by sol-gel route without using any precursor template are characterized by different morphology and phase composition. The films and the power show red and yellow luminescence with the most intensive photoluminescence (PL) bands at 612 nm and 588 nm, respectively. Raman, secondary ion mass spectrometry (SIMS), and X-ray diffraction (XRD) analysis of undoped nanostructured porous SrTiO_3_ films showed the presence of TiO_2_, SrO, and SrTiO_3_ phases and their components. The undoped porous SrTiO_3_ films are photosensitive and demonstrate resistive switching. The capacitance-voltage hysteresis loops with the width of about 6 V in the frequency range of 2 kHz—2 MHz were observed.

## 1. Introduction

Materials containing Eu^3+^ ions are promising as red phosphors in optoelectronic devices, e.g., LEDs and lasers, due to their excellent photoluminescence properties [1,2,3]. Optical radiation in the europium emission band at 612 nm is perceived by the human eye as red light, which makes these materials particularly important. The luminescence spectrum can differ in different crystal matrices. Material composition, crystal structure, and presence of defects have a significant influence. 

Oxide compounds with a perovskite structure, such as strontium titanate (SrTiO_3_), are widely used in nonlinear optics [4], electro-optical modulators [5], photocatalysis [6,7], thin-film capacitors [8,9], and memory devices [10,11]. It is a good host matrix for Eu^3+^ due to its radiation resistance and thermal stability.

Properties of SrTiO_3_ depend not only on the chemical composition, but also on the structure and morphology [12,13]. As a result of the modification of titanium and layered metal oxide systems, fabricated oxide coatings have different compositions, surface morphology, and properties that are determined by the structural parameters, in particular, nanosized grains [14,15], which is also right for more complex oxide systems. A decrease in the grain size of SrTiO_3_ at the nanoscale leads to distinctive properties as compared to the bulk material [16,17]. In recent years, several attempts were made to synthesize porous perovskites SrTiO_3_ and BaTiO_3_ [18,19].

Nanostructured perovskites have great potential for applications. Nanostructured SrTiO_3_ is a promising material for water purification from heavy metals [19] and photocatalysis [20]. Grain size and porosity affect the electrophysical parameters of perovskites. For example, porous barium titanate has a lower dielectric constant [21], and the Curie temperature increased up to 350 °C as compared to the bulk material [18]. Hysteresis of capacitance-voltage (C-V) characteristics of bulk monocrystralline SrTiO_3_ with Ni and Au electrodes was investigated before and after irradiation in [22,23], where C-V hysteresis was considered from the point of the drift of nonequilibrium charge carriers in the crystal. The hysteresis effects were observed not only in dielectric properties but also in magnetization loops [24,25]. Thus, the study of nanostructured porous perovskites is of particular interest for obtaining new properties of materials and creating novel structures based on them.

The synthesis of such nanostructured composites is possible by chemical methods, which include the sol-gel technology. This technology has a low cost, allows variation of the grain size, phase composition, concentration of dopants. The use of titanium alkoxides is the most promising for the synthesis of nanostructured SrTiO_3_, since they easily hydrolyze, forming hydrated oxides. By varying the temperature of dehydration, one can control the dispersion and morphology, the phase composition and the physical properties of the resulting xerogels. The use of metal alkoxides in the synthesis of multicomponent oxides ensures a high chemical homogeneity of the product, significantly reducing the temperature of oxide phases formation [26,27].

One of the synthesis techniques includes using templates, which control formation periodical porous SrTiO_3_ structure and repeatable combs form [28]. Another approach is based on using alcoxides and influence of water on the morphology of xerogels [18,29,30,31]. This approach allows for the fabrication of porous perovskites with grains larger than 100 nm. Nevertheless, the formation of porous perovskites with much smaller grains is a problem to be solved.

In this paper we present structural, luminescent and dielectric properties of porous undoped and Eu-doped SrTiO_3_ films with grains as small as 30 nm fabricated on monocrystalline silicon substrates by the sol-gel technique without any templates and compare them with those of identically synthesized bulk materials.

## 2. Materials and Methods

Porous SrTiO_3_ films doped with Eu, undoped ones and SrTiO_3_:Eu powder were synthesized using water containing sols. The sols were synthesized in the following way. First titanium isopropoxide was dissolved in a mixture of ethylene glycol monomethyl ether and nitric acid to prevent gelation initiated by titanium isopropoxide. Then, strontium nitrate was dissolved in the distilled water, followed by the addition of ethylene glycol monomethyl ether. Finally, both solutions were mixed to obtain the sols. To fabricate europium doped sols europium nitrate was added.

The sols were deposited by spinning at the rate of 2700 rpm for 30 s on monocrystalline (111) silicon wafers. The samples were then dried at 200 °C for 10 min and next film was identically deposited and dried. The deposition was repeated 5–10 times in order to fabricate the film of an appropriate thickness. Final calcination was performed at 800 °C for 40 min in air. Porous SrTiO_3_:Eu_x_ (x = 0.053) films containing 5 layers and undoped SrTiO_3_ films containing 10 layers were fabricated on silicon substrates. Powder materials were synthesized by the same heat processing of the precursors in a ceramic crucible.

Silicon substrate covered by TiO_x_/Pt layers with Pt playing a role of an isolated from the substrate electric contact was used for fabrication of the heterostructure Si/TiO_x_/Pt/SrTiO_3_/Ni for electrical measurements. The top round nickel electrode of about 330 µm in diameter was formed by ion-beam evaporation technique [9].

The morphology of the experimental samples was examined with scanning electron microscope S-4800 (Hitachi, Tokyo, Japan). The photoluminescence spectra were recorded at room temperature using a laser spectroscopic complex based on a Solar TII MS 7504i spectrograph-monochromator (Solar TII, Minsk, Belarus) in which a 1 kW xenon lamp was used for excitation. The photoluminescence light was detected by a digital camera (Proscan, Minsk, Belarus) with a cooled silicon CCD matrix. To isolate monochromatic lines from a wide spectrum of the lamp, we used a Solar TII DM 160 double monochromator (Solar TII, Minsk, Belarus). The photoluminescence was excited by the monochromatic light with the wavelength of 345 nm.

XRD (BOUREVESTNIK JSC, Saint Petersburg, Russia) studies of the films were carried out with a DRON-3 diffractometer using monochromatized CuKα radiation.

Raman spectra were measured with 3D Scanning Laser Confocal Raman Microscope Confotec NR500 (SOL Instruments, Minsk, Belarus) using 473 nm laser radiation for excitation. The measurements were performed at room temperature.

The depth distributions of the elements were investigated by secondary-ion mass spectrometry (SIMS) (TOF.SIMS 5, IONTOF, Munster, Germany). Secondary positive ions were produced by the bombardment of the samples with 30 keV Bi^+^ ions (1 pA). In situ etching of the samples was performed by sputtering with a focused 2 keV Cs^+^ ion beam (100 nA) raster scanned across a sample surface of 150 µm^2^.

Capacitance-voltage (C-V) and current-voltage (I-V) characteristics were recorded at room temperature using an Agilent E4980A Precision LCR meter (Agilent Technologies, Bayan Lepas, Pulau Pinang, Malaysia) integrated with Keithley 6517B (Keithley Instruments, Solon, Ohio USA) voltage source and digital V7-23 voltmeter, V7-27A ammeter and stabilized TEC-23 power supply, respectively. The C-V measurements were performed in the frequency range of 2 kHz–2 MHz. All measurements were done in automatic mode, implemented using the LabVIEW engineering graphic programming environment [32].

## 3. Results and Discussion

### 3.1. Porous Films and Powder Containing Strontium Titanate and Europium Ions

**Figure 1:**
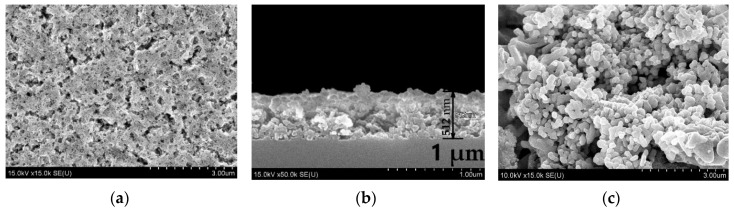
SEM images of the five-layer SrTiO_3_:Eu^3+^ film (**a**,**b**) and SrTiO_3_:Eu^3+^ powder (**c**).

The SEM images of the five-layer SrTiO_3_:Eu^3+^ film with the thickness of about 500 nm and identically synthesized powder are given in Figure 1. The films clearly reveal high porosity and highly developed surface. Grains in the powder are larger than in the film.

**Figure 2:**
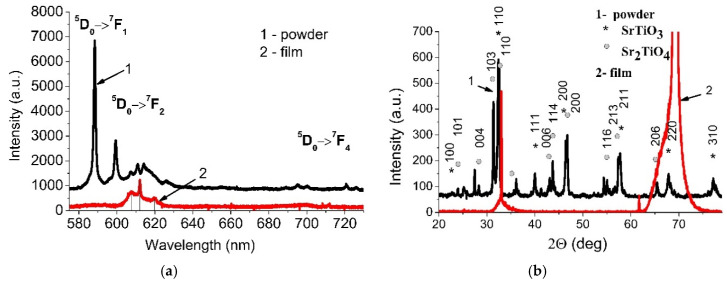
PL spectra of the SrTiO_3_:Eu^3+^ powder and five-layer SrTiO_3_:Eu^3+^ film (**a**) and diffraction patterns of the SrTiO_3_:Eu^3+^ powder and the SrTiO_3_ film (**b**).

PL spectra of the films presented in Figure 2a demonstrate the bands at 607, 612 and 619 nm corresponding to electrical dipole transition ^5^D_0_ → ^7^F_2_.

Due to the high solubility of europium ions and the ability to deploy in defective areas of the crystal, the incorporation of these ions can contribute to the phase formation in the material [33,34]. The intense PL band corresponding to the ^5^D_0_ → ^7^F_2_ electrical dipole transitions of Eu^3+^ indicates that europium occupies the position in the SrTiO_3_ crystallites that does not coincide with the symmetry center, most likely in defect regions [35].

PL spectra of the synthesized powder contain the bands at 588, 599, 607, 611, 626, 695, 700, 720 and 726 nm corresponding to the ^5^D_0_ → ^7^F_1_, ^5^D_0_ → ^7^F_2_, ^5^D_0_ → ^7^F_4_ transitions (Figure 2a). In this case the most intensive PL band is observed for magnetic dipole transition ^5^D_0_ → ^7^F_1_ at 588 nm. It indicates that Eu^3+^ ions occupy the positions with the symmetry center [35].

The XRD analysis of the powder presented in Figure 2b shows the diffraction peaks typical for SrTiO_3_ (PDF 01-089-4934) and Sr_2_TiO_4_ (PDF 00-039-1471). There are no signals belonging to the europium oxide. In addition to the identified peaks, there are ones that can be attributed to unreacted initial oxides and related solid solutions. The observed formation of Sr_2_TiO_4_ phase can be a result of Eu substitution by Sr leading to the enrichment of the powder with Sr.

XRD pattern of the five-layer SrTiO_3_:Eu^3+^ film contains the intense peak associated with the silicon substrate (Figure 2b). Apparently, the SrTiO_3_:Eu^3+^ film has a diffuse line in the region of 32.2 deg (influx to the left of the first sharp peak).

The powder synthesized from the same sol as the porous film has much larger grains as compared with the porous film and is characterized by the obvious phase formation. As a result, the PL spectra of the powder and the film differ. The presence of defects and disordering of the crystal structure certainly affects the luminescence spectrum of europium. The most intensive peak at 588 nm corresponding to the magnetic dipole transition ^5^D_0_ → ^7^F_1_ confirms that Eu^3+^ occupies the position with the symmetry center to be consistent with the XRD data. The typical doping concentration for lanthanides in solids is about 1 atomic %. In this case, it is not mandatory that trivalent lanthanide ion substitute only trivalent ion in host matrix, and there are no other local states for lanthanides. Luminescence of trivalent lanthanide ions with typical transitions between the corresponding terms was observed in inorganic perovskites: Tb^3+^ in YAlO_3_ [36], Er^3+^ in LiNbO_3_ corresponds to a [37], Eu^3+^ in BaTiO_3_ [38], as well as Eu^3+^ in sol-gel derived amorphous yttrium alumina composites [39]. Europium ion is the dopant that can exhibit divalent or trivalent valence state [40,41]. The luminescence spectra correspond to PL bands of Eu^3+^, as the PL spectra of Eu^2+^ lies in the region of about 380–500 nm [40]. In the case when Eu^3+^ occupies the position of strontium while remaining trivalent, the charge is compensated by the field of local environment, for example, titanium ions [42]. Replacement of divalent ions with trivalent leads to distortions of the crystal lattice of the structure [43,44]. Since the degree of doping of the image with europium is low; doping of the sample does not introduce significant distortions of the crystalline environment of europium ion, which corresponds to the presence of a narrow intense magnetic transition ^5^D_0_ → ^7^F_1_ (588 nm) in the spectrum. The electric dipole transition ^5^D_0_ → ^7^F_2_, observed at 599–626 nm, is allowed only when Eu^3+^ is located at a noncentrosymmetric crystallographic site. Otherwise considering low solid-solubility limit of Eu^3+^ in the perovskite matrix, such PL spectra is probably the result of the simultaneous exchange substitution of Sr^2+^ and Ti^4+^ positions by Eu^3+^ ions [42].

### 3.2. Heterostructures Si/SrTiO_3_/Ni and Si/TiO_x_/Pt/SrTiO_3_/Ni

**Figure 3:**
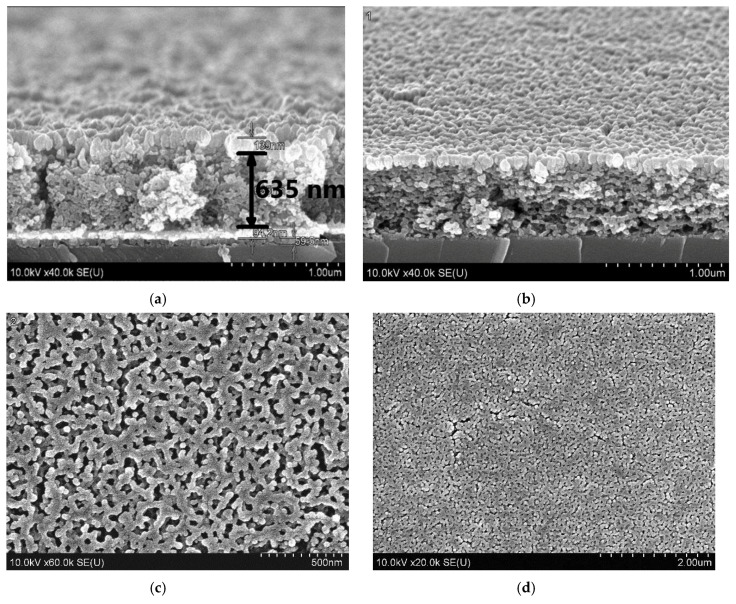
SEM images of Si/TiO_x_/Pt/SrTiO_3_/Ni (**a**), Si/SrTiO_3_/Ni (**b**) heterostructures, the surfaces of SrTiO_3_ (**c**) and Ni electrode (**d**).

Figure 3 shows cross-sections and plane views of Si/TiO_x_/Pt/SrTiO_3_/Ni and Si/SrTiO_3_/Ni heterostructures. The structures contain ten-layer porous SrTiO_3_ films with the total thickness of about 630 nm and the average grain size of about 30 nm.

**Figure 4:**
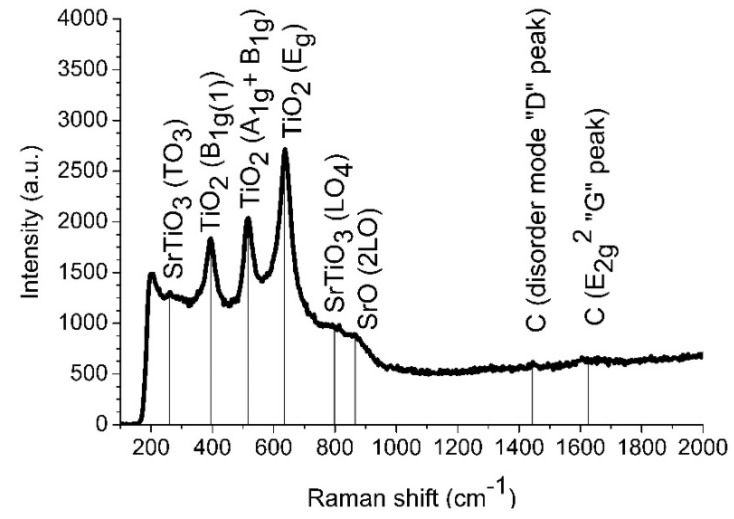
Raman shift spectra of Si/TiO_x_/Pt/SrTiO_3_/Ni heterostructure.

A Raman spectrum of the Si/TiO_x_/Pt/SrTiO_3_ heterostructure is presented in Figure 4. Several bands corresponding to TiO_2_ and SrTiO_3_ phases are observed.

Three bands at 395, 515 and 635 cm^−1^ correspond to TiO_2_ anatase crystalline phase. The band at 395 cm^−1^ corresponds to B1g vibration mode, the band at 635 cm^−1^ corresponds to Eg and the band at 515 cm^−1^ is a doublet of A1g and B1g modes [45]. The bands at 261 and 797 cm^−1^ are an indication of cubic SrTiO_3_ phase in the film. The band at 261 cm^−1^ is associated with first-order transversal TO_3_ phonon mode and the band at 797 cm^−1^ is a longitudinal LO4 phonon mode [46,47,48]. The activation of TO3 and LO4 bands indicates that structural distortions extended over a distance of the order of the phonon wavelength in the synthesized material [46]. Other SrTiO_3_ bands may be veiled by strong bands of TiO_2_. The single band at 865 cm^−1^ can be associated with SrO 2LO mode [49]. Two lines located at 1443 and 1625 cm^−1^ are related to carbon presenting in the structure [50]. A summary of the band identification is given in Table 1.

Porous films with small grains go through difficulties with the formation of the SrTiO_3_ phase in conditions of limiting grain growth because it is accompanied by the development of defects. They make TiO_2_ to dominate over SrTiO_3_.

**Figure 5:**
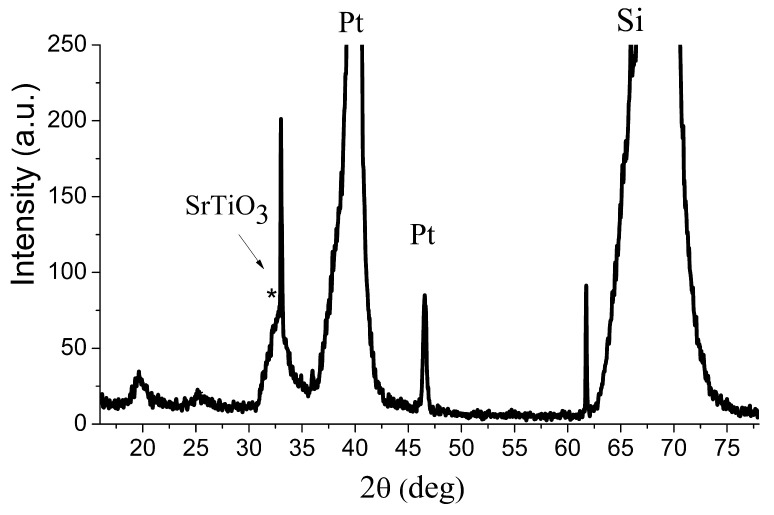
XRD spectra of Si/TiO_x_/Pt/SrTiO_3_/Ni heterostructure.

The XRD pattern of Si/TiO_x_/Pt/SrTiO_3_ heterostructure contains intense peaks associated with silicon substrate and platinum layer (Figure 5). The five-layer SrTiO_3_:Eu^3+^ film XRD pattern of Si/TiO_x_/Pt/SrTiO_3_ heterostructure contains a diffuse line in the region of 32.2 deg associated with SrTiO_3_. They also contain unidentified lines that can be attributed to oxides and oxide composites, including components of the precursors.

**Figure 6:**
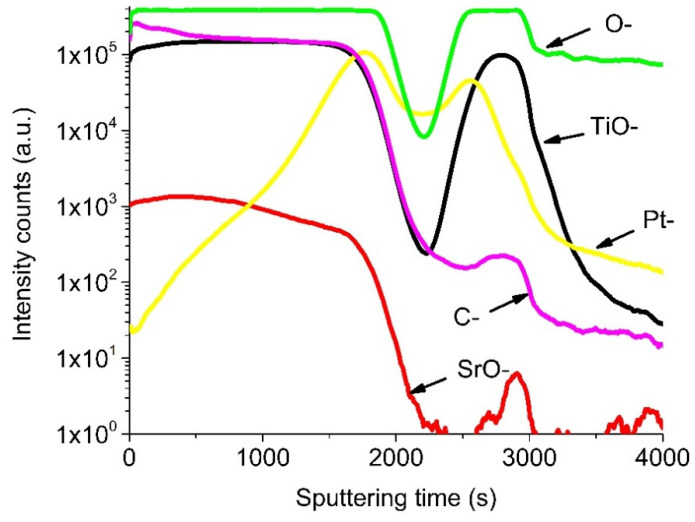
SIMS depth distribution of the precursor components in the Si/TiO_x_/Pt/SrTiO_3_ heterostructure.

Figure 6 shows SIMS depth distribution profiles of the components in the porous nanostructured SrTiO_3_ film formed on Pt/TiO_x_/Si substrate.

The thickness of the SrTiO_3_ film estimated from the SIMS data is about 450 nm. It is smaller than the one determined by SEM which is explainable noting that the sputtering rate of porous structures is larger than of dense films [51]. Qualitatively, the SIMS data are in a good agreement with the results of XRD and Raman spectroscopy analysis. TiO_2_, SrO, and SrTiO_3_ are formed homogeneously in the nanostructured porous films.

The SIMS depth profiles of the components in the Si/TiO_x_/Pt/SrTiO_3_ heterostructure illustrate the presence of several layers. The layer corresponding to the sputtering time of 1800 s is enriched with SrO we associate with the porous SrTiO_3_. It contains some carbon that is a residue of the incomplete elimination of the carbon containing isopropoxy groups and organic groups of the solvent [51,52]. The next layers corresponding to the sputtering time in the ranges of 1800–2500 and 2500–3000 s are associated with the Pt and TiO_x_, respectively. Silicon ions of the substrate were not detected under these experimental conditions.

**Figure 7:**
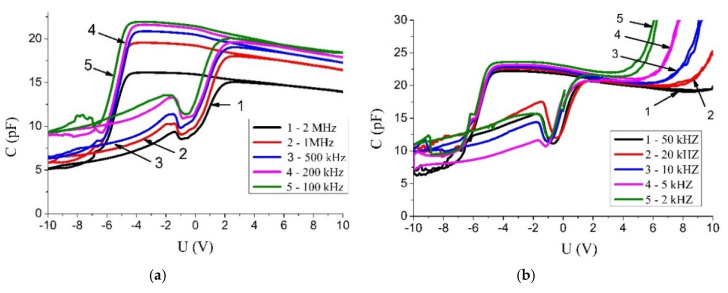
Capacitance-voltage characteristics of the Si/SrTiO_3_/Ni heterostructure recorded in the frequency ranges of 100 kHz-2 MHz (**a**) and 2 kHz-50 kHz (**b**).

Figure 7 shows C-V characteristics of the Si/SrTiO_3_/Ni heterostructure after several measurement cycles at different frequencies. Initially, the structure was in the state with the maximum capacity followed by the high frequency bypass performed from 0 V counterclockwise. The voltage polarity corresponds to the sign of the electric potential on one top electrode relative to the other top electrode. The charge of the capacitor basically occurs only by applying a positive voltage. The capacity of this structure with two upper electrodes varies from 6 pF to 22 pF. The width of the hysteresis is ~6 V in the frequency range of the test measurement signal from 2 kHz to 2 MHz. The width of the hysteresis loop is about 6 V.

The nonlinearity of the C–V characteristics is caused by the presence of electrically active deep energy levels in the band gap, created by various defects. As the capacity growth is observed only with one positive polarity, electrons are trapped by the defect states [22]. The nonlinear dependence of the capacitance on voltage applied can be associated with the formation of potential wells in the surface layer of nanostructured grains due to the porosity.

The frequency dependence of the hysteresis loop area indicates the presence of fast and slow energy states involved in the recharging of electrical capacitance. A sharp increase of the capacitance at a positive bias voltage with a decrease in the frequency of the test signal to 2 kHz indicates the electrical charge accumulation on slow energy states. A shift of the C-V characteristics towards negative bias voltages by approximately the same value indicates the presence of a fixed positive charge in the SrTiO_3_ film or near the interfaces. The nature of the traps and energy states is still not clear and may be the goal of a separate study by electric thermally stimulated methods.

**Figure 8:**
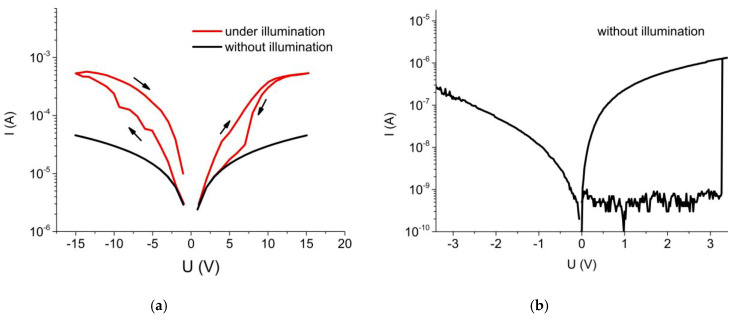
In-plane (**a**) and transversal (**b**) I-V characteristics of Si/SrTiO_3_/Ni (**a**) and Si/TiO_x_/Pt/SrTiO_3_/Ni (**b**) containing ten layers of SrTiO_3_ under and without illumination.

In-plane I-V measurements between two top Ni electrodes of the structure Si/SrTiO_3_/Ni show that illumination of the samples with the light of a halogen lamp at the intensity of 39 mW/cm^2^ leads to significant changes in the forward and reverse branches of the I-V curves. This is illustrated in Figure 8a. Under illumination, the curves are characterized by hysteresis for both forward and reverse biasing, which was also early observed in [53]. The transversal electrical measurements on the Si/TiO_x_/Pt/SrTiO_3_/Ni structure shows resistance switching even without illumination (Figure 8b) when the bias was applied between top Ni and bottom Pt electrodes.

## 4. Conclusions

We described the sol-gel approach to the fabrication of porous Eu doped SrTiO_3_ multilayer film structures. The technique does not require any organic template precursors in the sol for making the material porous. Both porous nanostructured films and bulk powders demonstrate the most intensive PL bands at 612 nm and 588 nm, respectively. Thus, this material can be used as correspondingly yellow and red phosphors in optoelectronic devices depending on the structural state of Eu^3+^ ions.

XRD, Raman spectroscopy, and SIMS analysis are in a good agreement with each other and confirm the formation of TiO_2_, SrO, and SrTiO_3_ phases and their components in the porous SrTiO_3_ material. The thin film structures, including porous SrTiO_3_, exhibit hysteresis of C-V and I-V characteristics, which is of interest for nonvolatile memory elements.

## Figures and Tables

**Table 1:** Raman spectra bands.

Peak, cm^−1^	Material	Mode	Reference
261	SrTiO_3_ (cubic)	TO3 (O–Sr–O bending)	[46,47,48]
395	TiO_2_ (Anatase)	B1g	[45]
515	TiO_2_ (Anatase)	A1g + B1g	[45]
635	TiO_2_ (Anatase)	Eg	[45]
797	SrTiO_3_ (cubic)	LO4 (Ti–O stretching)	[46,47,48]
865	SrO	2LO	[49]
1443	Carbon	Disorder	[50]
1625	Carbon	E2g	[50]
